# Machine Learning-Based Analysis in the Management of Iatrogenic Bile Duct Injury During Cholecystectomy: a Nationwide Multicenter Study

**DOI:** 10.1007/s11605-022-05398-7

**Published:** 2022-07-05

**Authors:** Victor Lopez-Lopez, Javier Maupoey, Rafael López-Andujar, Emilio Ramos, Kristel Mils, Pedro Antonio Martinez, Andres Valdivieso, Marina Garcés-Albir, Luis Sabater, Luis Díez Valladares, Sergio Annese Pérez, Benito Flores, Roberto Brusadin, Asunción López Conesa, Valentin Cayuela, Sagrario Martinez Cortijo, Sandra Paterna, Alejando Serrablo, Santiago Sánchez-Cabús, Antonio González Gil, Jose Antonio González Masía, Carmelo Loinaz, Jose Luis Lucena, Patricia Pastor, Cristina Garcia-Zamora, Alicia Calero, Juan Valiente, Antonio Minguillon, Fernando Rotellar, Jose Manuel Ramia, Cándido Alcazar, Javier Aguilo, Jose Cutillas, Christoph Kuemmerli, Jose A. Ruiperez-Valiente, Ricardo Robles-Campos

**Affiliations:** 1 Department of Surgery and Transplantation, Virgen de La Arrixaca Clinic and University Hospital, Murcian Institute of Biosanitary Research (IMIB), Ctra. Madrid-Cartagena, 30120 s/nEl Palmar, Murcia, Spain; 2 Department of Hepatobiliary Surgery and Transplants, Hospital Universitario La Fe, Valencia, Spain; 3 Department of Hepatobiliary Surgery and Liver Transplant Unit, Hospital Universitari Bellvitge, University of Barcelona, Barcelona, Spain; 4 Department of Information and Communication Engineering, Universidad de Murcia, Murcia, Spain; 5 Hepatobiliary Surgery and Liver Transplant Unit, Cruces University Hospital, Bilbao, Spain; 6 Department of Surgery, Hospital Clínico, University of Valencia, Biomedical Research Institute INCLIVA, Valencia, Spain; 7 Department of Surgery, Hepatopancreatobiliary Unit, Hospital Clínico San Carlos, Madrid, Spain; 8 Department of Surgery, Morales University Hospital, Madrid, Spain; 9 Department of Surgery, Alcorcon University Hospital, Madrid, Spain; 10 Department of Surgery, Miguel Servet University Hospital, Saragossa, Spain; 11 Hepatobiliopancreatic Surgery Unit, Hospital de La Santa Creu I Sant Pau, Universitat Autònoma de Barcelona, Barcelona, Spain; 12 Department of Surgery, Los Arcos del Mar Menor University Hospital, Murcia, Spain; 13 Department of Surgery, General University Hospital, Albacete, Spain; 14 Department of General Surgery, Digestive Tract and Abdominal Organ Transplantation, Hospital Universitario, 12 de Octubre, Madrid, Spain; 15 Department of Surgery, Puerta del Hierro University Hospital, Madrid, Spain; 16 Department of Surgery, Reina Sofía University Hospital, Murcia, Spain; 17 Department of Surgery, Rafael Mendez Hospital, Murcia, Spain; 18 Department of General Surgery, Elche University Hospital, University Miguel Hernández of Elche, Alicante, Spain; 19 Department of General Surgery, Hellin Hospital, Albacete, Spain; 20 Department of General Surgery, Obispo Polanco Hospital, Teruel, Spain; 21 HPB and Liver Transplant Unit, Department of General Surgery, Clinica Universidad de Navarra, Universidad de Navarra, Pamplona, Spain; 22 Institute of Health Research of Navarra (IdisNA), Pamplona, Spain; 23 HPB Surgery and Liver Transplantation, Dr. Balmis General University Hospital, and Alicante Institute for Health and Biomedical Research (ISABIAL), Alicante, Spain; 24 Department of General Surgery, Hospital Lluís Alcanyís Hospital, Xàtiva, Valencia Spain; 25 Department of General Surgery, Hospital Francesc de Borja, Gandía, Valencia Spain; 26 Department of Surgery and Transplantation, University Hospital Zurich, Zurich, Switzerland; 27 Department of Surgery, Clarunis - University Centre for Gastrointestinal and Liver Diseases, Basel, Switzerland

**Keywords:** Iatrogenic bile duct injury, Cholecystectomy, Machine learning, Artificial neural network

## Abstract

**Background:**

Iatrogenic bile duct injury (IBDI) is a challenging surgical complication. IBDI management can be guided by artificial intelligence models. Our study identified the factors associated with successful initial repair of IBDI and predicted the success of definitive repair based on patient risk levels.

**Methods:**

This is a retrospective multi-institution cohort of patients with IBDI after cholecystectomy conducted between 1990 and 2020. We implemented a decision tree analysis to determine the factors that contribute to successful initial repair and developed a risk-scoring model based on the Comprehensive Complication Index.

**Results:**

We analyzed 748 patients across 22 hospitals. Our decision tree model was 82.8% accurate in predicting the success of the initial repair. Non-type E (*p* < 0.01), treatment in specialized centers (*p* < 0.01), and surgical repair (*p* < 0.001) were associated with better prognosis. The risk-scoring model was 82.3% (79.0–85.3%, 95% confidence interval [CI]) and 71.7% (63.8–78.7%, 95% CI) accurate in predicting success in the development and validation cohorts, respectively. Surgical repair, successful initial repair, and repair between 2 and 6 weeks were associated with better outcomes.

**Discussion:**

Machine learning algorithms for IBDI are a novel tool may help to improve the decision-making process and guide management of these patients.

**Supplementary Information:**

The online version contains supplementary material available at 10.1007/s11605-022-05398-7.

## Introduction

Iatrogenic bile duct injury (IBDI) is a major complication of cholecystectomy. While the technique for laparoscopic cholecystectomy (LC) has improved over the years, the incidence of IBDI remains unchanged due to miss interpretation of normal hepatobiliary anatomy. In particular, vascular hepatic lesions that show involvement of the intrahepatic bile duct are associated with higher morbidity and mortality such that a liver transplant is required in some cases.^1–3^ The incidence of IBDI during cholecystectomy is influenced by the anatomy of the patient, nature of the pathology, intraoperative complications, such as bleeding, and technical errors.^4,5^

IBDI is associated with multiple sequelae that require multiple therapeutic interventions. As such, it must be managed well to achieve successful results and minimize complications.^6,7^ At the onset, IBDI must be diagnosed immediately, and the patient must be referred to a specialized center without delay.^8,9^ The long-term prognosis of IBDI is also dependent on the skill of the surgeon performing the repair, and the approach selected for the repair. Among these factors, early detection of IBDI and its sequelae are the keys to proper management.^10^ However, IBDI is poorly documented. Most of the studies on IBDI provide incomplete data on the severity of the condition and fail to identify all of the sequelae involved. The Comprehensive Complication Index (CCI) is a potential tool that can summarize all of the postoperative complications associated with IBDI.^11^ The CCI uses a metric approach to quantify complications.

Several algorithms have been developed to aid in the medical decision-making process.^12–15^ Once machine learning reaches a certain threshold, the technology can be applied to improve IBDI management. Our study aimed to design a decision tree model to identify the factors that influence the success of the initial repair, as well as develop a risk-scoring system based on the CCI data. The quality of both models was validated using an independent cohort of patients.

## Methods

### Study Design

This study was a retrospective multi-institution cohort study. Patients diagnosed with IBDI in 22 Spanish centers between January 1990 and July 2020 were included in our study. Ethical approval for this study was obtained from the Clinical Research and Ethics Committee at the Virgen de la Arrixaca Clinic and University Regional Hospital Murcia, Spain (Internal Protocol Code: 2021–5-3HCUVA). All the centers completed a uniform data collection protocol with clear variable definitions.

### Variable Selection

The initial dataset contained 40 variables with data on 748 patients. We collected data on the demographic variables, clinical characteristics, and clinical aspects of each patient. The demographic variables included age and gender. The clinical characteristics included the type of approach (open or laparoscopic), intraoperative diagnosis, days to diagnosis, whether the hospital was specialized, whether surgical repair was required, whether the repair was performed in the same hospital, and the time to repair (less than 2 weeks, between 2 and 6 weeks, or more than 6 weeks). For the second model, we also included data on whether the initial repair was successful. The clinical aspects of the patient included the American Society of Anesthesiologist (ASA) scores (≥ 3), whether the patient had risk factors, whether it was a type E IBDI, and whether the patient had any vascular injury.

Definitive repair was defined as success when the goal of injury repair was reached by endoscopy, interventional radiologist or surgery. Success of the repair included the removal of drains, stent-free, the absence of symptoms of bile leak infection or the absence of signs of obstruction (cholestasis, cholangitis or stenosis). We defined specialized centers as those that performed liver transplantation or performed complex hepatobiliary surgery and only referred those patients who required liver transplantation.

IBDI was classified according to Strasberg’s classification. The patient risk factors analyzed included cholecystitis, cholangitis, bleeding, scleroatrophic gallbladder carcinoma, anatomical abnormalities, Mirizzi syndrome, choledocholithiasis, and acute or chronic pancreatitis. The primary intervention included surgical, radiologic, or endoscopic procedures. The surgical repair techniques included primary suturing, end-to-end anastomosis, cystic repair, use of the Kehr tube, lavage plus drainage, Roux-en-Y hepaticojejunostomy, liver resection, or liver transplantation. The radiological approaches included percutaneous drain and stent placement. The endoscopic interventions included stent placement and papillotomy.

### Decision Tree and CCI-Based Risk Score Models Construction

We proposed two different models in this study. The first model examined the success of the initial repair (yes or no) by using a recursive conditional inference tree with the *ctree* package in R. We then incorporated the CCI into this model. The CCI weighs the severity of all postoperative complications according to the Clavien–Dindo classification and expresses morbidity through a continuous numeric scale from 0 (no complications) to 100 (death).

The second model examined the effect of risk groups on the prognosis of IBDI repair. We grouped CCI scores into ranges and characterized each range as being low (0–25), low-medium (25–50), medium–high (50–75), and high (75–100) risk. We grouped these scores together because we expected little differentiation in the level of risk within each range. We then implemented a random forest regression model using the *caret* package in R to obtain the predicted CCI values for our study population. As such, we were able to stratify patients according to their level of risk.

The hyperparameters of both models were finetuned by implementing tenfold cross-validation, which highlighted the values that provided the best quality metrics while avoiding overfitting. Statistical significance was set at *p* < 0.05, and statistical analysis was performed using R programming language (Version 3.6.1; R Foundation for Statistical Computing, Vienna, Austria).

### Validation and Quality Metrics

To validate the generalizability of our models, we excluded the data from several hospitals during the development of the model. As such, both models were developed with the data from 15 hospitals (596 patients) (development cohort) and validated with the data from seven hospitals (152 patients) (validation cohort).

We determined the quality metrics for the development and validation cohorts. Our first model was a binary model. We evaluated this model using classical binary classifier metrics, which included the area under the receiver operator characteristic curve (AUC), accuracy, sensitivity, specificity, *kappa*, F1-score, and precision. The second model was evaluated as follows: we initially performed a regression analysis to determine the root mean square error (RMSE), mean absolute error (MAE), and R-squared values. We then classified our data into the aforementioned CCI ranges and verified this using accuracy, *kappa*, and the confusion matrix.

## Results

**Fig. 1:**
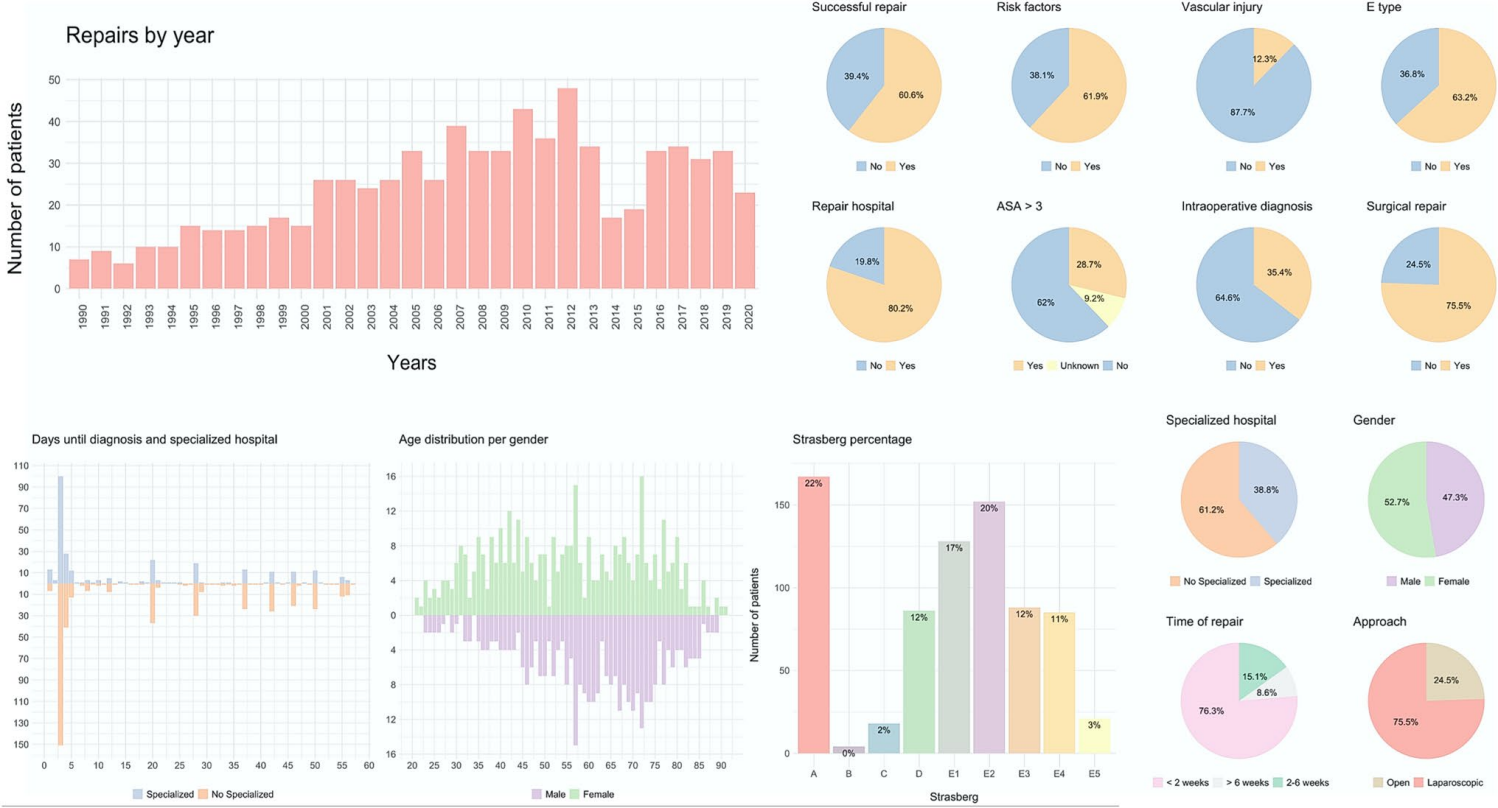
Chronological evolution of the number of cases for each year collected by the participating centers and a summary of the main variables used for the development and validation of both models

We analyzed 748 patients across 22 hospitals in Spain. Our study population comprised 394 (52.7%) women and 354 (47.3%) men. The mean age of the patients in this cohort was 57 ± 16.72 years. Figure 1 shows the distribution of the variables. The mean CCI of patients was 46.1 (± 19) with a 4.9% of mortality. A total of 15 patients (2%) required a liver transplant. The rate of success according to the need for a first, second, third or fourth repair attempt was 60.6, 78.3, 68.2, and 72.7%, respectively. The development cohort included data on 596 patients from 15 hospitals across 12 provincial administrative regions. In contrast, the validation cohort included data on 152 patients from seven hospitals across five provincial administrative regions.

### Decision-Making Model on the Success of the Initial Repair

**Fig. 2:**
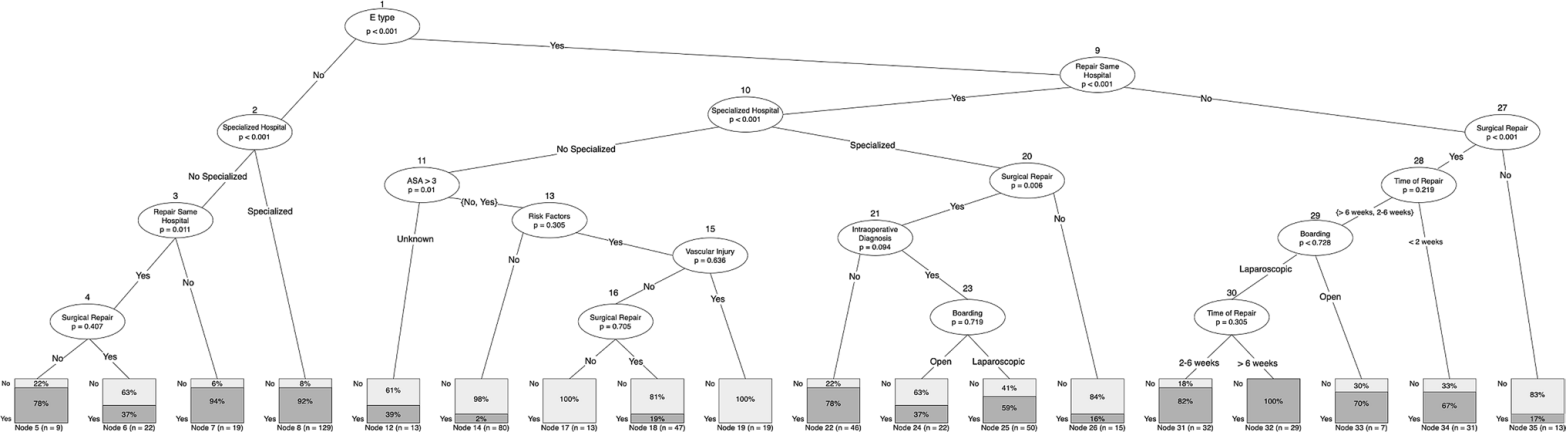
Base-tree model on the success of the initial repair. The unbiased regression tree includes all the variables listed in the base model in the text. The maximum depth of the tree is set at four layers for simplicity. The higher a variable appears in the tree, the more predictively important the variable was lesion Type E. Decision making according lesions non type E (**a**) and type E (**b**)

This model provides a visual interpretation of the patients who demonstrated good results because their IBDI were adequately managed, irrespective of whether the initial repair was successful (Fig. 2). Some examples of the interpretation of this decision tree are as follows: a patient with type A–D IBDI who was managed in a specialized center has a higher probability of a successful initial repair (nodes 7–8) than if the initial repair was performed in a non-specialized center (nodes 5–6). In contrast, a patient with type E IBDI has a better prognosis if the primary intervention was surgical (nodes 31–34 and 35 for surgical and non-surgical repair, respectively). However, our model also demonstrated that the success rate fell when the repair was done early because the need for early or emergency repair was associated with more severe IBDI. Our model also demonstrated that patients with type E IBDI fared better with treatment in specialized centers (nodes 22, 24–26), but the failure rate increased if these specialized centers did not perform surgical repair (node 26). For cases that were treated in non-specialized centers (nodes 12, 14, and 17–19), the success rate dropped significantly if the patient had risk factors or vascular problems. The most important variables in this model (Table 1) were the type of injury (E type) (*p* < 0.001), whether the hospital was specialized (*p* < 0.001), whether the repair was performed in the same hospital where the IBDI occurred (*p* < 0.001), and whether the repair was surgical (*p* < 0.001).

**Table 1:** Distribution of the variables included in the decision model related with the probability of initial repair success

Variable	Total Data No. (%)	Development cohort No. (%)	Validation cohort No. (%)	*p* value
Demographic characteristic				
Age, mean (SD)	57 (16.72)	57.6 (16.41)	57 (17.38)	0.53
Men	354 (47.3%)	278 (46.6%)	76 (50.0%)	0.39
Clinical characteristic				
Open approach	183 (24.5%)	137 (22.9%)	46 (30.2%)	0.05
Laparoscopic approach	565 (75.5%)	459 (77.0%)	106 (69.7%)	0.05
Intraoperative Diagnosis	265 (35.4%)	220 (36.9%)	45 (29.6%)	0.02
Days to diagnosis, mean (SD)	18.66 (18.21)	18.22 (18.1)	20.36 (18.34)	0.01
Specialized Hospital	290 (38.8%)	262 (43.9%)	28 (18.4%)	< 0.001
No specialized Hospital	458 (61.2%)	334 (56.1%)	124 (81.6%)	< 0.001
Repair Same Hospital	600 (80.2%)	464 (77.8%)	136 (89.4%)	< 0.001
Surgical Repair	565 (75.5%)	468 (78.5%)	97 (63.8%)	< 0.001
Time of Repair < 2 weeks	571 (76.3%)	435 (72.9%)	136 (89.4%)	0.08
Time of Repair 2—6 weeks	113 (15.1%)	101 (16.9%)	12 (7.8%)	0.08
Time of Repair > 6 weeks	64 (8.6%)	60 (10.2%)	4 (2.6%)	0.08
Success first repair	453 (60.6%)	326 (54.7%)	127 (83.5%)	**
Patient characteristic				
ASA > 3	215 (28.7%)	172 (28.8%)	43 (28.2%)	< 0.001
Risk Factors	463 (61.9%)	368 (61.7%)	95 (62.5%)	0.084
E type	473 (63.2%)	417 (69.9%)	56 (36.8%)	< 0.001
Vascular injury	92 (12.3%)	86 (14.4%)	6 (3.9%)	0.21

Among patients in the development cohort, our quality metrics analysis demonstrated that this model was 81% (range, 77.8–91.1%; 95% confidence interval [CI]) accurate in predicting whether the initial repair would be successful. Additionally, the model had a sensitivity and specificity of 0.92 and 0.76, respectively. We then tested this model on the validation cohort. Among patients in the validation cohort, this model was 82.8% (range, 0.76–0.89; 95% CI) accurate in predicting the success of the initial repair. However, the specificity and sensitivity of the model decreased to 0.87 and 0.47, respectively. The quality metrics of this model are listed in Supplementary Table 1.

### Risk-Scoring Model Based on the CCI

**Fig. 3:**
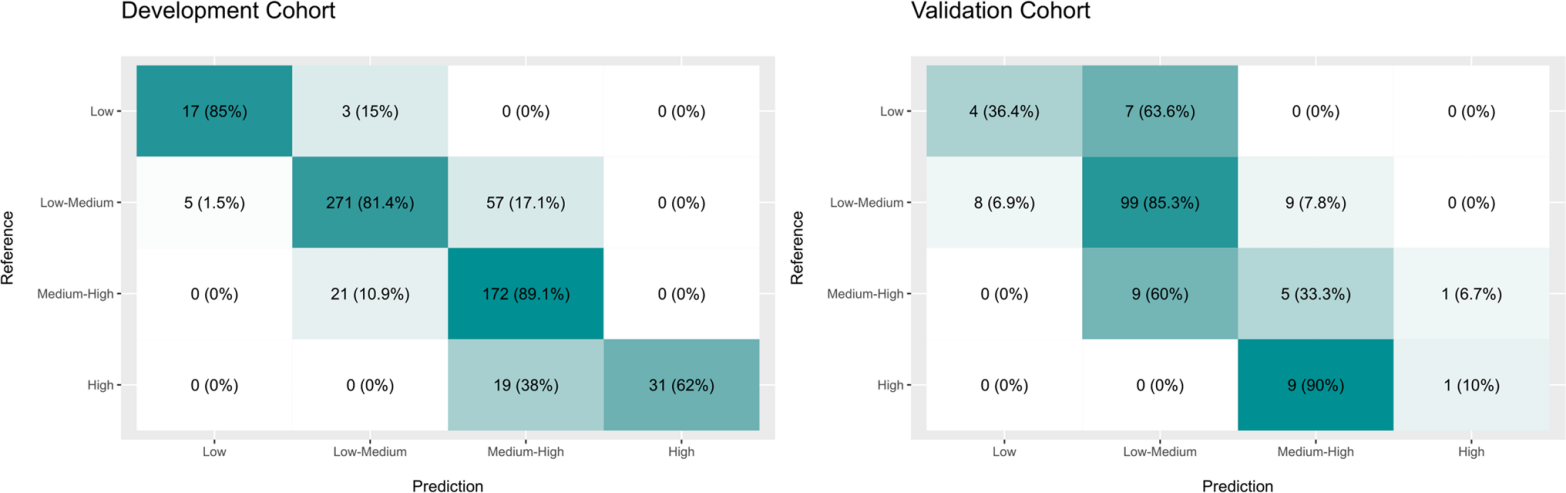
Confusion matrix for CCI ranges. We observed the dynamics of the model predictions and which cases are more frequently confused. In each cell we observe the absolute number of cases that fall there, and also the percentage that number represents in the row. The intensity of the color of the cell represents that percentage. Notice that the diagonal of the matrices represents the same reference and prediction, therefore the true prediction rate of each category. Moreover, we see that those predictions that are wrong, mostly fall in the subsequent category, therefore, the errors might be considered as not large and they might be partially influenced by the sampling of each bucket

The second model was based on the CCI, which utilizes the Clavien–Dindo classification to score risk from 0 to 100. We built a random regression forest model that predicted the potential patient’s CCI. We applied this model to the development cohort, which resulted in MAE and RMSE values of 5.67 and 7.65 points, respectively. We also applied this model to the validation cohort, which resulted in MAE and RMSE values of 9.70 and 13.13 points, respectively. The predicted CCI scores were classified into ranges that corresponded to different risk categories. Applied to the development and validation cohorts, the model was able to predict the CCI range in 82.3% (range, 79.0–85.3%; 95% CI) and 71.7% (range 63.8–78.7%; 95% CI) of cases, respectively. Figure 3 shows the confusion matrix for the CCI ranges. The quality metrics analysis of this model is listed in Supplementary Table 2.

Our random forest analysis demonstrated that the most important variables in this model were whether the initial repair was successful, whether the initial repair was surgical, whether the repair was performed between 2 and 6 weeks of the diagnosis, and whether the repair was performed at the same hospital as the diagnosis. The remaining variables are listed in Table 2.

**Table 2:** Importance of each of the variables used in the development and validation of the CCI risk-score model

Variable	Variable Importance Model 2
Demographic characteristic	
Age, mean (SD)	31.43
Men	–
Clinical characteristic	
Open approach	–
Laparoscopic approach	11.25
Intraoperative Diagnosis	33.14
Days to diagnosis, mean (SD)	36.85
Specialized Hospital	–
No specialized Hospital	22.05
Repair Same Hospital	42.69
Surgical Repair	80.64
Time of Repair < 2 weeks	–
Time of Repair 2—6 weeks	48.13
Time of Repair > 6 weeks	14.84
Success first repair	100.0
Patient characteristic	
ASA > 3	24.10
Risk Factors	35.89
E type	31.02
Vascular injury	33.83

## Discussion

This is the first study that analyzes on the largest cohort of patients in the literature with IBDI the potential of artificial intelligence models in their management. Our study provided two sets of results because we analyzed two models. We initially implemented a decision tree analysis centered on the success of the initial repair and then proposed a risk-scoring model based on the CCI. Both models demonstrated that patients diagnosed with IBDI who are managed in specialized hospitals and with surgical repair achieve the best outcomes and the least rate of complications. In addition, it also shows that not only those more serious injuries, but also minor injuries must be referred to specialized centers to improve results.

Machine learning algorithms offer a decision-making procedure to help solve complex problems. Decision-making process after a IBDI continues to be complex due to every patient and scenario are different and there are several factors that influence the correct decision. Machine learning algorithms compared to traditional logistic regression models or Cox proportional hazards models are more actionable and interpretable. In addition, these computational models usually have much better performance than traditional logistic regressions as they can fit the data better. The use of artificial intelligence models has the special advantage of their ability to analyze in more detail the meaning or nature of complex or imprecise data, to extract patterns and detect trends than with other commonly used modeling approaches. Commonly used models in medical literature are often parametric, like the linear regression or a naïve bayes, and therefore are limited by the low flexibility capabilities of the model. This is not the case with the non-parametric models used in this study, that include decisions trees and random forest.

These models have deeper flexibility to fit the data as they do not have the parametric constraints of the other models. Therefore, these models can be effectively used by surgeons as part of the decision process when they need to assess the next step of a treatment. For example, given the demographic, type of IBDI, clinical and patient characteristics, the surgeon board can insert into the model different procedures to analyze the success of the repair, or the need to refer to a specialized hospital, obtaining repair success probabilities of those potential decisions based on the historical data fitting the models. Analogously, this can also be done to obtain a risk factor based on the CCI model. Moreover, is important to denote that this model is not static, meaning that as time goes by, new data can be included to recalculate the model, facilitating a continuous improvement in the management of iatrogenic bile duct injury over time. Finally, we should take into account that the model is only taking into account a subset of the characteristics that can affect the success of the surgery, but many others, like the emotional status of the patient or the specific ability of the surgeon are not taken into account. Therefore, the final decision needs to be taken holistically based on all the evidence and knowledge gathered by the surgeon, instead of solely relying on the model probabilities.

Recent consensus measures have been proposed to minimize the risk of IBDI in LC,^7,16,17^ due to nowadays, IBDI remains a problem of great clinical and quality of life significance.^18^ The incidence of IBDI in this present national registry has remained more or less stable since 2000. IBDI may occur due to a number of reasons. In particular, we noted risk factors in 61.9% of our study population; IBDI occurred most frequently in the setting of acute cholecystitis (68.4%), scleroatrophic gallbladder (14.4%), and pancreatitis (4.3%).

Two of the most important variables identified in our models were related to the capacity of the hospital where the primary surgery was performed and whether the repair was performed in the same hospital. While 35.4% of the IBDI in this series were diagnosed intraoperatively, most cases were diagnosed at an average of 18 days after the cholecystectomy, with some IBDI diagnosed even years after. In addition, also showed that most non-specialist IBDI repairs that were performed by the same surgeon responsible for the injury had very low success rates and an increased risk of mortality. Immediate repair performed by an experienced surgical team and in the absence of sepsis provided the best long-term results.^19,20^

Our analysis also demonstrated that type E IBDIs were statistically associated with poorer outcomes. Strasberg–Bismuth types E4 and E5 IBDI are often associated with vascular lesions.^21^ In our series, 33.3% of types E4 and E5 IBDI were associated with vascular lesions. A published series on IBDI demonstrated that concomitant injury to the hepatic artery was observed in 12–40% of patients, especially with the laparoscopic approach.^22^ These patients also usually have a history of repeated bilioenteric anastomosis procedures and/or multiple stents and have recurrent cholangitis and/or chronic cholestasis, which increases their risk for secondary biliary cirrhosis, hepatic artery lesions, and eventually fulminant liver failure.^23^

Our risk-scoring model and random forest analysis also demonstrated that it was important whether the initial repair was successful and whether the primary intervention was surgical. As such, the greater the number of failed attempts until definitive repair, the greater the risk of developing postoperative complications and recurrent stenosis. This was also demonstrated by a large retrospective study published by Reuver et al..^24^ A multidisciplinary approach that includes therapeutic endoscopy and interventional radiology is essential for the proper management of IBDI.^25–28^ However, endoscopic or percutaneous interventions should only be considered as an alternative to surgical repair in type A IBDI that do not have strictures and are not associated with anatomical abnormalities. In contrast, endoscopic treatment of more complex lesions, such as stenosis, is associated with failure in 30–59% of cases and higher re-stenting rates. Overall, Roux-en-Y hepaticojejunostomy remains the gold standard for the reconstruction of damaged bile ducts during cholecystectomy.^29,30^

We also classified the predicted CCI values into the low (0–25), low-medium (25–50), medium–high (50–75), and high (75–100) risk categories. Our model performed significantly worse in the validation cohort, which may also be attributed to differences between the hospitals in both cohorts. Our confusion matrix provided some insight. In Fig. 3, each cell indicates the absolute number of cases and its corresponding percentage, the more intense the color of the cell, the higher the corresponding percentage. The diagonal cells in our matrices have the same references and predictions, which indicate that they represent the true prediction rate of each category. Moreover, the incorrect predictions from our model fall into the neighboring categories; therefore, the value of the error is likely to be small and only partially influenced by the sampling of each risk range. The matrices also demonstrate that the high-risk category may be problematic because it has the lowest positive prediction rate in both cohorts.

**Fig. 4:**
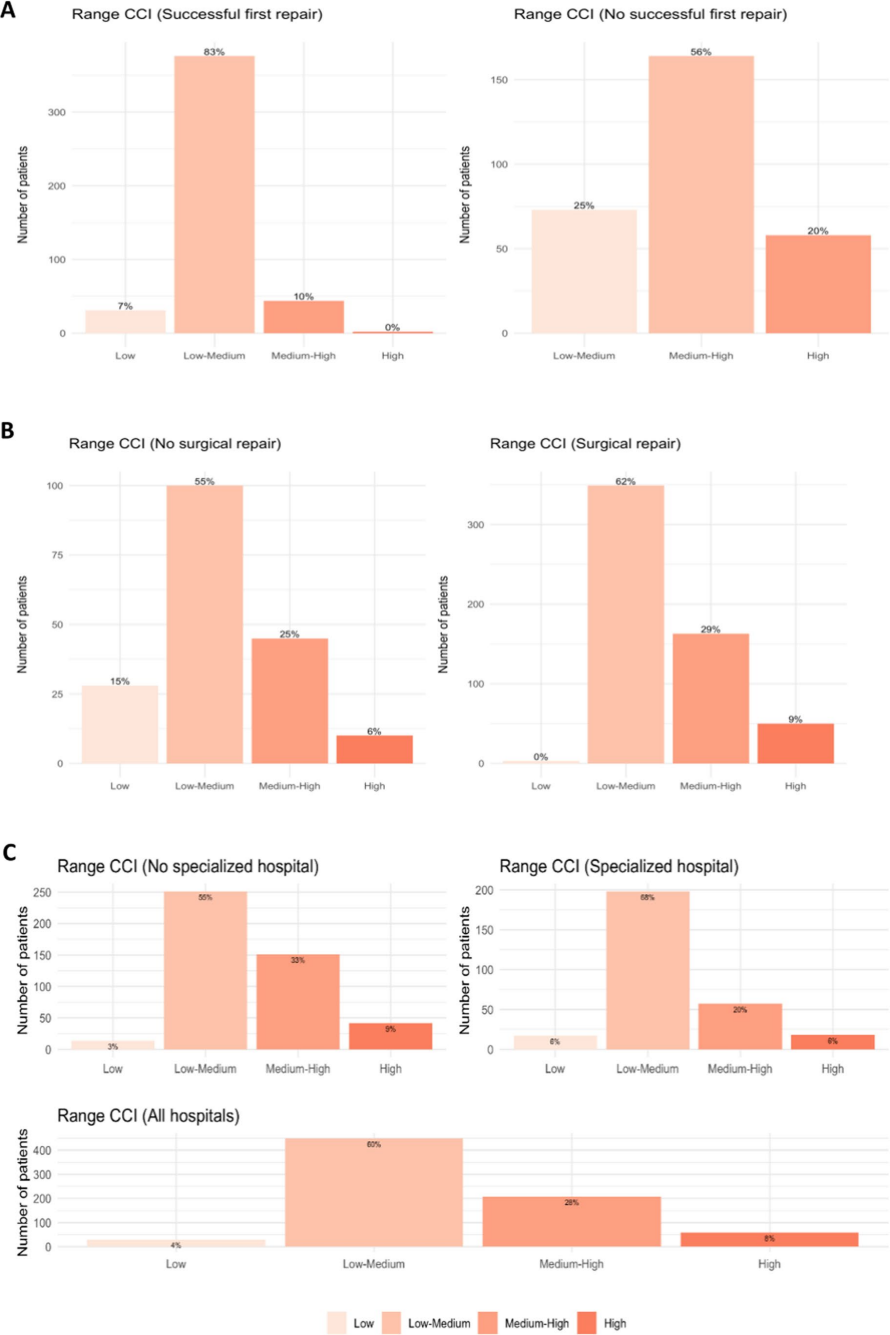
Visualizations of the distribution CCI ranges for successful of firs repair (**a**), surgical repair (**b**), and specialized or non-specialized hospitals (**c**)

We further analyzed the variables identified by our random forest analysis. First, we assessed if there was an association between the CCI range and success of the initial repair. We found low CCI ranges among patients whose initial repair was successful (Fig. 4a), which was expected. Second, we assessed if there was an association between the CCI range and type of surgical repair. We knew from the first model that surgical repair increases the likelihood of a successful initial repair. However, the second model demonstrated that surgical repair was observed more often in the higher CCI ranges (Fig. 4b). We expect worse outcomes with higher CCI ranges. However, this observation was because patients who needed surgical repair tended to have more severe IBDI. Therefore, while the surgical repair technique was better, we found higher CCI ranges because these patients had more severe IBDI at the onset, in addition to possible complications during repair, which would have also increased their CCI scores. Third, we assessed for an association between the CCI range and level of hospital specialization and found that there were more patients from the medium–high and high CCI groups in non-specialized centers (33% and 9%, respectively) than in specialized centers (20% and 6%, respectively) (Fig. 4c).

Our study present certain limitations. The specificity of our model decreased when we applied the model to the new set of patients in the validation cohort. This may be due to our methodology because we did not analyze whether the hospitals in the development and validation cohorts were similar. There may have been differences in terms of the level of specialization, methods, and data collection in the hospitals between both cohorts, which affected our model’s predictive ability. However, the decrease in the MAE and RMSE values was not significant. On the other hand, the data for this study come from many institutions over a highly evolved period of time with a retrospective data source, which can be associated with a significant amount of data loss, bias, and precision. Storage of patient’s information was not as accurate in the nineties compared to contemporary data, and this happens in most of the specialized centers if data is not collected prospectively. Even so, we have included the graph because it is representative of how cases increased with the introduction of laparoscopy and how the percentage of injuries, although it has suffered a decrease, remains more or less stable. Finally, there is also a limitation about the general applicability of these complex modeling systems especially in community settings where many of these injuries occur and initial management may be delayed or misdirected. This delayed care and often poor initial therapeutic intervention may make ultimate outcomes worse even if this machine learning is utilized as a tool once referred to a specialty center. The development of a risk calculator where the surgeon could enter the characteristics of the patient and evaluate the probability of success of the possible decisions could help to generate a treatment plan, especially when decision making is based on the surgical judgment of a seasoned biliary surgeon.

## Conclusion

Management of IBDI injuries is a difficult multidisciplinary task, with heterogeneous results around the world as it includes many issues to consider. Therefore, it is almost impossible to develop prospective randomized controlled trials. Many of the conclusions have been obtained over time through case series. The development of an algorithm and a risk scoring model represents a new approach based on learning matching that aims to improve the results in these patients while being aware of the treatment of these patients. Our analysis stood out above other factors that delays in definitive repair (i.e., due to attempts at non-surgical repair) and delayed specialized center remission resulted in poorer outcomes. Furthermore, not only patients with complex type E lesions but also those with less severe lesions will benefit the most from being referred to a specialized center as soon as possible to begin the decision-making process. Our risk-scoring model can then be used to estimate the patients’ level of risk based on the initial presentation and IBDI type. The patient’s risk category may be used to determine the appropriate management for IBDI. Surgical departments can benefit from these models because they are based on the actual historical data and can aid in decision-making. As data are further collected and generated through the models, the models can be continuously improved, as well.

## Supplementary Information

Below is the link to the electronic supplementary material.

Supplementary file1 (DOCX 16 KB)

## Abbreviations

ASA: American Society of Anesthesiologist
AUC: Area under curve
CCI: Comprehensive Complication Index
IBDI: Iatrogenic bile duct injury
LC: Laparoscopic cholecystectomy
MAE: Mean absolute error
RMSE: Root mean square error
