# Supplementary material for: Machine Learning-Based Analysis in the Management of Iatrogenic Bile Duct Injury During Cholecystectomy: a Nationwide Multicenter Study
Source: J Gastrointest Surg. 2022 Jul 5;26(8):1713–23. doi: 10.1007/s11605-022-05398-7 (PMC9439981; doi:10.1007/s11605-022-05398-7)
Supplement: Supplementary file 1 — Supplementary file1 (DOCX 16 KB) [file 11605_2022_5398_MOESM1_ESM.docx]

**SUPPLEMENTARY INFORMATION**

Table S1. Quality Metrics Model 1

| **Metrics** | **Development Cohort** | **Validation Cohort** |
| --- | --- | --- |
| Accuracy (95% CI) | 0.810 (0.776 - 0.841) | 0.828 (0.759 - 0.885) |
| Sensitivity | 0.919 | 0.470 |
| Specificity | 0.760 | 0.874 |
| Kappa | 0.607 | 0.285 |
| Precision | 0.637 | 0.320 |
| F1 | 0.752 | 0.380 |
| AUC | 0.795 | 0.624 |

Table S2. Quality Metrics Model 2

| **Metrics** | **Development Cohort** | **Validation Cohort** |
| --- | --- | --- |
| RMSE | 7.65 | 13.13 |
| MAE | 5.67 | 9.70 |
| R squared | 0.85 | 0.52 |
| Accuracy (95% CI) | 0.823 (0.790 - 0.853) | 0.717 (0.638 - 0.787) |
| Kappa | 0.693 | 0.294 |
